# Mediators of the association between low socioeconomic status and poor glycemic control among type 2 diabetics in Bangladesh

**DOI:** 10.1038/s41598-020-63253-8

**Published:** 2020-04-21

**Authors:** Mosiur Rahman, Keiko Nakamura, S. M. Mahmudul Hasan, Kaoruko Seino, Golam Mostofa

**Affiliations:** 1 Department of Global Health Entrepreneurship, Division of Public Health, Graduate School of Medical and Dental Sciences, Tokyo Medical and Dental University, Tokyo, Japan; 2 Department of Population Science and Human Resource Development, University of Rajshahi, Rajshahi, 6205 Bangladesh; 3 Department of Environmental Health Sciences, School of Public Health, The University of Michigan, Michigan, USA

**Keywords:** Type 2 diabetes, Lifestyle modification, Risk factors

## Abstract

Although low socioeconomic status (SES) is related to poor glycemic control, the underlying mechanisms remain unclear. We examined potentially modifiable factors involved in the association between low SES and poor glycemic control using data from the baseline survey of a multicenter, prospective cohort study. Five hundred adult type 2 diabetes patients were recruited from three diabetes centers. Glycemic control was poorer in diabetic individuals with low SES than in those with higher SES. Adverse health-related behaviors, such as non-adherence to medication (adjusted odds ratio [AOR] = 1.07, 95% confidence interval [CI] 1.04–1.13) and diet (AOR = 1.04, 95% CI 1.02–1.06); existing comorbidities, such as depressive symptoms (AOR = 1.05, 95% CI 1.04–1.09); and non-adherence to essential health service-related practices concerning diabetes care, such as irregular scheduled clinic visits (AOR = 1.04, 95% CI 1.03–1.06) and not practicing self-monitoring of blood glucose (AOR = 1.05, 95% CI 1.03–1.07), mediated the relationship between social adversity and poor glycemic control specially in urban areas of Bangladesh. Those identified factors provide useful information for developing interventions to mitigate socioeconomic disparities in glycemic control.

## Introduction

There is a great deal of evidence that glycemic control reduces many of the long-term complications of diabetes, especially microvascular complications, making it an important goal of diabetes care^1,2^. However, glycemic control remains an elusive goal for many patients with type 2 diabetes worldwide. Bangladesh has a diabetes rate of 10.8% among adults, only 13.0% of them showed appropriate control of blood glucose level^3^. Moreover, the burden of diabetes and its rate of control are not equally distributed among populations in this country. Both the prevalence and control of diabetes showed marked inequality across age, socioeconomic, and educational groups^3^.

Socioeconomic status (SES) is associated with inequality significantly in both the prevalence and control of diabetes, and its associations can potentially be modified. In Bangladesh, as in many other developing countries, individuals with low SES have poorer glycemic control than those with higher SES^3,4^, leading to more complications of their disease, including a higher mortality rate. While low SES and poor glycemic control are known to be related^3–6^, little is known about the underlying mechanisms.

The association between low SES and poor glycemic control is probably as a result of three sets of mediating variables such as adverse health-related behaviors, comorbid conditions, and non-adherence to essential health service-related practices. Evidence shows that, optimal glycemic control can be attained in people with type 2 diabetes through rigorous patient self-management of various health-related behaviors, including avoiding tobacco use, taking medication, following a diet, and regular physical activity, corresponding to recommendations from a health care provider^7^. Furthermore, health-related behaviors tend to differ according to SES. There is an extensive body of research investigating the relationship between SES and adverse health-related behaviors in both developed^8–11^ and developing countries^12–14^. Results found that low SES is associated not only with tobacco use^12^, but also with physical inactivity^8,13^, nonadherence to medication^9,14^, and an unhealthy diet^10,11^. However, in contrast to developed countries^8^, leisure time physical activity in Bangladesh^13^ is more prevalent in the lower SES group. Thus, adverse health-related behaviors might mediate the relationship between low SES and poor glycemic control.

Among the comorbid conditions, being overweight/obese was generally associated with an increased likelihood of poor glycemic control in type 2 diabetics^15^. Studies also noted that comorbid conditions such as hypertension^16^ and depressive symptoms^17^ are correlated with poorer glycemic control. Besides, hypertension, obesity, and depressive symptoms often exhibit socio-economic patterning^18,19^, low prevalence of hypertension and overweight/obesity^18^ and high prevalence of depressive symptoms^19^ were observed in the low SES group. Therefore, these comorbid conditions may potentially mediate the relationship between low SES and poor glycemic control.

Apart from adverse-health related behaviors and comorbid conditions, non-adherence to essential health service-related practices including irregular scheduled visits to diabetes clinic, not practicing self-monitoring of blood glucose concentrations, and reliance on alternative medicine have been known to contribute to poor glycemic control^14,20–22^. Several observational studies found that underuse of recommended preventive services associated with poor glycemic control^20,21^. Studies also indicated that patients who were not self-monitored their blood glucose^14^ and rely on alternative medicine^22^ had higher odds of having poorly controlled blood glucose compared to those who self-monitored their blood glucose and do not rely on alternative medicine. Furthermore, these essential health service-related practices vary by SES. In comparison to the high-SES group, people in the low-SES group are more likely to use low-cost and often less-effective alternative medicines and to experience barriers in the timely use of health care services according to their need^23^. Accordingly, non-adherence to essential health service-related practices has been proposed as potential mediators of the association between low SES and poor glycemic control.

To our knowledge, there has been only a single study regarding to examine the mediators contributing to socioeconomic inequality in glycemic control^24^, which showed that avoidance coping during a stressful event related to their diabetes (eg, to give up trying to deal with the event or to refuse to believe it is happening) and depressive symptoms mediated the relationship between SES and glycemic control. However, the contributions of mediators relating low SES to poorer glycemic control may differ according to context, suggesting the need for research in different settings. To date, there have been no studies in developing countries to assess the mediators relating low SES to poorer glycemic control. Therefore, evidence on socioeconomic disparities in glycemic control and potential mediators to mitigate this relationship is required.

This study was performed to examine potential modifiable factors (i.e., adverse health-related behaviors, comorbidities, and non-adherence to essential health service-related practices concerning diabetes care) involved in the association between low SES and poor glycemic control and to elucidate the extent to which these factors explained the association between low SES and poor glycemic control as mediators among adult diabetes patients in Bangladesh.

## Methods

### Design and settings

This study was performed using data from the baseline survey of the multicenter, prospective cohort “Bangladesh Diabetes study,” which was performed between August 2018 and September 2018. Type 2 diabetic patients were recruited from specialized outpatient diabetes clinics in hospitals located inside metropolitan areas and affiliated with the Diabetic Associations of Bangladesh (DAB), a nonprofit association of several hospitals and health centers across the country that is mostly responsible for diabetes care in Bangladesh. The selected hospitals are located in metropolitan areas and provided primary to tertiary care services to patients coming from both rural and urban areas. Assuming poor glycemic control among 18.4% of type 2 diabetic patient’s adherent to medication^25^, to achieve a power of 80%, a level of significance of 5%, and detect a prevalence ratio of poor glycemic control of 1.60^25^, the target sample size for medication-non-adherent vs. medication-adherent patients was 470. The sample size was further increased to 500 to account for attrition.

From each center, the base cohort was assembled by selecting type 2 diabetic patients aged 21 years and older. In addition to age, the other inclusion criteria were permanent residents in the study locations, diagnosis of diabetes for at least 1 year, and at least one prescription claimed for antidiabetic medication during the patient selection period. Patients with previous insulin prescription, which may indicate more advanced disease, and women with a history of polycystic ovarian syndrome or a diagnosis of gestational diabetes were excluded.

Participants selection was performed in three stages:

Stage 1: Diabetes centers were chosen from three of the seven divisional cities in Bangladesh, i.e., one from Dhaka, one from Rajshahi, and one from Barishal. The inclusion criteria for selection of these centers were: (i) administrative support and approval from these centers; (ii) previous experience of the research team with these centers; (iii) availability of interviewers experienced in clinical research; (iv) laboratories and centers were in the same locations or in close physical proximity; and (v) availability and proximity of eligible participants.

Stage 2: One center from each of the selected divisional cities was then selected by simple random sampling.

Stage 3: 500 patients were finally selected from these three centers through equal proportion: Dhaka 168; Rajshahi 166; and Barisal 166.

### Data collection

At baseline, consecutive patients with type 2 diabetes from the participating centers who fulfilled the eligibility criteria were recruited through face to face communication and were invited to participate in the study. Each participant received a study manual summarizing the details of study visits and measurements. Baseline data collection was performed through a structured questionnaire, blood tests, blood pressure and anthropometric measurements, and other procedures. The questionnaires were drafted in English and then translated into Bangla, the national language of Bangladesh.

### Data quality assurance

Data quality was assured by adequate design, and the questionnaire was pre-tested on 10% of the entire sample (n = 50) that were not included in the survey. The baseline survey was administered after amendment of ambiguities identified in the questionnaires. The data collectors and supervisors were given 2 days of intensive training to familiarize themselves with the tools and methods of data and blood specimen collection. The collected data were checked carefully for completeness, accuracy, and clarity every day by a supervisor, and the principal investigator monitored the overall activities of data collection. One interviewer and one medical laboratory technologist together with the principal investigator were involved in data collection at each center. We examined the reliability and internal consistency of the dietary and physical activity questionnaire data based on Cronbach’s α coefficient; the Cronbach’s α were 0.82 and 0.85 for the dietary and physical activity instruments, suggesting that the level of internal consistency was high. With regard to reproducibility, the two sets of responses from the patients in the test–retest group were inspected using the intraclass correlation coefficient. For example, the intraclass correlation coefficient for the physical activity questionnaire was 0.90, indicating satisfactory test–retest reliability.

### Measurements

#### Mediators

**Figure 1:**
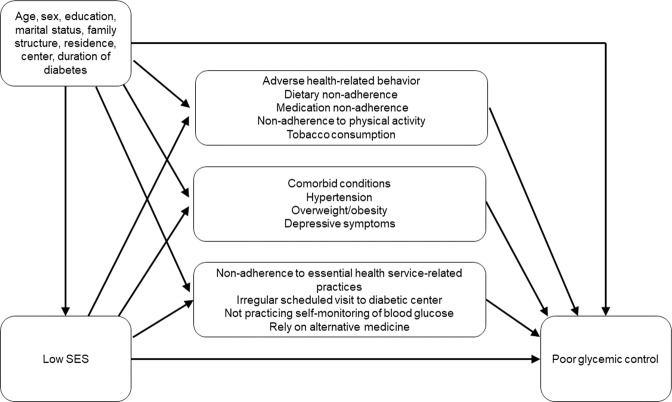
Pathways assumed for the domains of mediators between low socioeconomic status (SES) and poor glycemic control.

The conceptual model underlying the present study suggests that there are three sets of mediating variables representing the mechanisms underlying the association between low SES and poor glycemic control: adverse health-related behaviors, comorbid conditions, and non-adherence to essential health service-related practices concerning diabetes care (Fig. 1).

*Health-related behaviors*. Adherence to oral antidiabetic medication(s) was calculated based on the omitted doses prescribed by the physician during the week before the interview according to the patient as follow: [(prescribed doses − omitted doses) × 100/prescribed doses]. A patient was considered adherent to antidiabetic medication(s) if the proportion was ≥80% of the prescribed dosage, as used in previous studies^26,27^.

Ten questions adapted from the Summary of Diabetes Self-care Activities measure^28^ and modified according to the nutritional guidelines for Bangladeshi adult diabetics^29^ were used to assess patients’ adherence to prescribed dietary regimens over the last 7 days. These questions elicited responses relevant to overall adherence to dietary instructions, recommended three meals per days, recommended servings of fruits and vegetables, consumption of low glycemic index carbohydrate-containing foods, high-sugar foods, high-fiber foods, n-3 fatty acids, healthy (monounsaturated) oils, sugary drinks, and high-fat foods, and were scored on a 7-point Likert scale with a maximum score of 70. A patient was considered adherent to dietary recommendations if the calculated total score was ≥80% of the possible total score (≥56).

Leisure time physical activity of the participants was measured using the translated Bangla version of the International Physical Activity Questionnaire (IPAQ). The validity of these physical activity questions in the Bangladeshi adult population was discussed in detail elsewhere^30^. Data on physical activity were assessed as the total number of minutes spent weekly doing moderate (e.g., conventional walking, bicycling with light effort, gardening, light exercises, e.g., cleaning house, involvement in games with children) to vigorous physical activities (e.g., running, jogging/running, bicycling with greater effort, fast swimming, team sports, e.g., football, volleyball, or basketball), and this measure was calculated separately for each activity. All adults should engage in at least 150 min/week of moderate-intensity or 75 min of vigorous-intensity physical activity or an equivalent combination of these. A patient was considered adherent to the recommended physical activity if [moderate physical activity + vigorous physical activity × 2] ≥ 150 minutes in 7 days^31^.

Tobacco use status was self-reported, and participants were classified as tobacco users if they currently smoke any tobacco products, such as cigarettes, cigars, or pipes, or currently use any smokeless tobaccos which are commonly available in Bangladesh, such as zarda, sadapata, gul, or snuff.

*Comorbid conditions*. This study used the OMRON HAM-8731 Blood Pressure Monitor (Omron Healthcare, Kyoto, Japan). The means of three different systolic and diastolic blood pressure readings measured at 5-minute intervals were used to report the respondents’ blood pressure values. Hypertension was defined as systolic blood pressure ≥ 140 mm Hg or diastolic blood pressure ≥ 90 mm Hg or use of antihypertensive medication^32^.

The validated Bangla version of the short form of the Depression, Anxiety, and Stress Scale (DASS-21)^33^ was used to evaluate depressive symptoms. Participants rated the extent to which they have experienced symptoms over the previous week on a 4-point Likert scale ranging from 0 (did not apply to me at all) to 3 (applied to me very much, or most of the time). The cut-off scores for depressive symptoms were developed according to the DASS manual: normal (0–9), mild (10–13), moderate (14–20), severe (21–27), and extremely severe (≥28). Participants were considered to suffer from depressive disorders if they scored ≥ 10.

Standing height was measured against a flat wall at the end of the expiration using a ruler pressed against the head crown and a measuring tape. Weight was measured in light cloths with bare-foot using a digital scale (WPCS-DS810 model). Body mass index (BMI) was determined as weight (kg) divided by the square of height (m^2^), with BMI ≥ 25 classified as overweight/obese^34^.

*Non-adherence to essential health service-related practices concerning diabetes care*. Compliance to regular clinical visits to their doctors was evaluated according to patients’ self-reporting to interviews. We created a binary variable, dichotomized as either visiting the center regularly, as per the schedule (1) or not (0). Practice of blood glucose self-monitoring was evaluated according to patients’ self-report to interviews. A binary variable was also designed to assess whether the patients self-monitored their blood sugar levels at home or at a drug store other than at the diabetes center (1) or not (0). To obtain information about whether the patients were reliant on alternative medicine, they were asked “Other than the diabetes center do you usually go for Homeopathic/Ayurvedic treatment? Answers were coded as “yes” or “no”.

### SES measurement

While other SES indicators, including education and occupation, capture individual-based dimensions of social position, household wealth index is more indicative of the standard of living and access to goods and services^35^, so the household wealth index was used as the primary measure of SES in the present study. The process of constructing the wealth index involves assigning wealth scores by principal component analysis based on 23 selected household assets, e.g., number of household members, floor, wall, and roof materials, type of cooking fuel, refrigerator, motorcycle, and others. Productive and non-productive rural-related assets (such as tractors and plough or axe, etc.) were also included, in addition to livestock (cows, goats, etc.), to prevent an urban bias. Variables with prevalences <3–5% (e.g., sources of drinking water, access to electricity, mobile phone, and cart) were excluded from the analysis in creating the wealth index^35^. Based on their weighted wealth scores, households were then divided into terciles; each tercile was given a rank, from 1 (poor) to 3 (rich), and respondents were ranked with regard to the total score of the household in which they lived.

### HbA1c measurement

HbA1c, an indicator of glycemic control, was the dependent variable in our analyses. Participants were asked to come into the designated diagnostic center after an overnight fast for ≥8 hours. Fasting venous blood was collected between 08:00 and 11:00 for measurement of HbA1c. A 3-ml venous blood sample was drawn from all participants into tubes containing sodium fluoride, and plasma was separated into new tubes and analyzed within 1 hour of collection. HbA1c was measured by high-performance liquid chromatography (D-10 HPLC Analyzer; Bio-Rad, Hercules, CA). The same laboratories in each center involved in the measurement of HbA1c were committed to participating in external quality affirmation schemes, assuring that laboratories with equivalent equipment could produce results that were comparable to each other. In accordance with the American Diabetes Association (ADA) guidelines, HbA1c < 7% was taken to indicate good glycemic control, while HbA1c ≥ 7% indicated poor glycemic control^36^.

### Covariates

This study also included the following sociodemographic and health-related factors: respondents’ age (21–46 years, 47–55 years, or 56–85 years), sex (male vs. female), educational status (no education, elementary, secondary, higher secondary and above), family structure (nuclear vs. joint) currently married (yes or no), center (Rajshahi, Barishal, or Dhaka), place of residence (urban vs. rural), and duration of diabetes (<5 years or ≥5 years).

### Statistical analyses

We estimated the age- and sex-adjusted percentages of people with diabetes with good glycemic control by a direct standardization method. We used the percentage of people with diabetes in our study sample according to age-sex structure as the standard population. Pearson χ2 tests were used to determine the statistically significant differences in sociodemographic, health-related, comorbid conditions, and essential health service-related behavioral characteristics in achieving good glycemic control. Differences in the distribution of potential mediators by SES were tested with the Mann–Whitney U test. Multivariable relationships between SES, potential mediators, and good glycemic control were explored using logistic regression analysis. To evaluate mediation of binary poor glycemic control outcome, we fitted the binary outcome version of the Baron and Kenny method^37^. The Stata command *binary_mediation* was used to compute the direct, indirect, and total effects in the applied model.

The direct effect is defined as influence of exposure on the outcome that is not mediated by designated potential mediators. The indirect effect answers the counterfactual question: were we to hold socioeconomic conditions constant at the lower socioeconomic condition, what change would occur in the level of exposure to mediators, e.g., non-adherence to medication in diabetic patients from the value realized under high socioeconomic conditions to the value realized under low socioeconomic conditions. To test mediation with a dichotomous outcome (poor control or not), we used logistic regression models to analyze the influence of binary exposures (low SES vs. high) on each mediator (binary), adjusted by covariates. The results are presented as AORs with 95% CIs created from 500 bootstrapped samples. As adherence to physical activity and proportions of non-overweight/obesity and non-hypertensive subjects were higher in participants with low SES than in participants with high SES in our study, the inclusion of these mediators in the regression equation may estimate inconsistent mediation (negative sign). Therefore, these variables were not considered in the regression model. The proportion of the indirect effect mediated by the mediators was calculated using the following formula:

$${\rm{Proportion}}\,{\rm{mediation}}=\,\log ({\rm{AORIE}})/\,\log ({\rm{AORTE}}){\rm{n}}\times {\rm{n}}100 \% $$

where IE represents the indirect effect and TE represents the total effect. All statistical analyses were conducted using Stata version 14 (Stata Corp, College Station, TX).

### Ethical considerations

Approval was obtained from the Ethical Review Committee of Tokyo Medical and Dental University, Japan (approval number, M2018-063, dated July 05, 2018) and by the research ethics boards of the Institute of Biological Sciences, University of Rajshahi, Bangladesh (approval number, 107/320/IAMEBBC/IBSc, dated November 08, 2018). Permission was obtained from the corresponding diabetic clinics to conduct the study. Informed consent for study participation was obtained from all study participants. The participants provided written consent to the study after receiving information about the objective of the study, collection of blood specimen for measurements of blood glucose and Hb1Ac, risks and benefits of being in a research study, and the confidentiality of the data. All the study procedures were conducted in accordance with the principles of the Declaration of Helsinki as revised in 2013. With consent, patients were offered to provide free medical care or payment if they experienced an unlikely event of any injury during blood specimen collection.

## Results

Table 1 shows the age- and sex-adjusted prevalence of achievement of good glycemic control, and the sociodemographic, health-related, comorbid conditions, and essential health service-related practices among diabetics. Of the total 500 patients, 28.2% showed good glycemic control, while significant proportion of patients (71.8%) showed poor glycemic control. Good glycemic control was significantly higher among participants in the high-SES group than the low-SES group (*P* < *0.00*1). At α = 0.05, the power of the above test is 0.980, which implies that, if SES and good glycemic control were indeed related to the extent suggested by the data in Table 1, the test would be able to detect that 98% of the time.

**Table 1:** Descriptive statistics of sociodemographic, health-related behaviors, comorbid conditions, and essential health service-related behavioral characteristics according to glycemic control among diabetes individuals: Barriers to diabetes control and periodontal disease study (*n* = 500).

Characteristics	n (%)	% (95% CI)^*1*^	*P*-value	Power
		Achievement of good glycemic control^*2*^		
**Socio-demographic**				
**Age, years**				
21–46 47–55 56–85	169 (33.8) 164 (32.8) 167 (33.4)	31.4 (13.1–49.7) 26.8 (6.8–46.9) 26.4 (13.6–39.1)	0.530	0.270
**Sex**				
Female Male	249 (49.8) 251 (50.2)	30.9 (15.0–46.9) 25.5 (8.6–42.4)	0.178	0.345
**Education**				
No education Primary Secondary Higher secondary and above	73 (14.6) 155 (31.0) 131 (26.2) 141 (28.2)	10.9 (3.4–25.2) 17.9 (5.9–41.8) 35.8 (29.4–42.2) 42.1 (19.2–65.1)	<0.001	0.989
**Types of family structure**^***3***^				
Nuclear Joint	400 (80.0) 100 (20.0)	25.3 (6.7–43.9) 38.8 (19.9–57.8)	0.007	0.805
**Currently married**				
No Yes	44 (8.8) 456 (91.2)	10.2 (2.2–37.7) 30.1 (14.3–45.9)	0.003	0.995
**Center**				
Rajshahi Barishal Dhaka	166 (33.2) 166 (33.2) 168 (33.6)	34.3 (32.6–48.7) 28.3 (26.0–41.6) 22.0 (19.6–34.2)	0.044	0.653
**Residence**				
Rural Urban	184 (36.8) 316 (63.2)	16.1 (4.9–27.3) 35.1 (13.7–56.5)	<0.001	0.997
**Socioeconomic status**				
Low Medium High	168 (33.6) 167 (33.4) 165 (33.0)	17.4 (8.0–34.0) 26.2 (18.2–34.3) 41.7 (20.2–63.2)	<0.001	0.980
**Duration of diabetes, years**				
<5 ≥5	249 (49.8) 251 (50.2)	37.4 (22.4–52.5) 18.5 (3.7–33.3)	<0.001	0.997
**Health-related behaviors**				
**Adherence to medication**				
No Yes	287 (57.4) 213 (42.6)	13.4 (1.4–25.3) 48.7 (31.5–65.9)	<0.001	0.998
**Adherence to diet**				
No Yes	370 (74.0) 130 (26.0)	17.6 (4.0–31.1) 58.7 (29.4–87.9)	<0.001	0.996
**Adherence to physical activity**				
No Yes	311 (62.2) 189 (37.8)	21.0 (9.8–32.2) 41.1 (16.4–65.7)	<0.001	0.993
**Tobacco consumption**^***4***^				
No Yes	434 (86.8) 66 (13.2)	30.3 (12.9–47.7) 12.2 (9.7–23.4)	0.005	0.902
**Comorbid conditions**				
**Hypertension**^***5***^				
No Yes	281 (56.2) 219 (43.8)	33.4 (17.6–49.2) 22.6 (3.4–41.7)	0.011	0.791
**Overweight/Obesity**^***6***^				
No Yes	295 (59.0) 205 (41.0)	29.3 (12.7–45.9) 26.8 (12.2–41.4)	0.570	0.128
**Depressive symptoms**				
No Yes	287 (57.4) 213 (42.6)	38.6 (18.9–58.3) 13.8 (2.7–24.9)	<0.001	0.998
**Essential health service-related practices**				
**Regular scheduled visit to diabetic center**				
No Yes	336 (66.8) 166 (33.2)	17.2 (6.5–28.2) 51.7 (32.9–70.4)	<0.001	0.998
**Practicing self-monitoring of blood glucose**				
No Yes	309 (61.8) 191 (38.2)	14.9 (6.0–24.0) 49.8 (29.2–70.5)	<0.001	0.998
**Rely on alternative medicine**				
No Yes	458 (91.6) 42 (8.4)	30.3 (15.0–45.5) 6.7 (2.0–11.4)	0.002	0.990
**Prevalence**		28.2 (24.4–32.3)		

Note: CI: Confidence interval ^1^Age-sex adjusted prevalence ^2^ Good glycemic control: HbA1c < 7%; ^3^Nuclear family: a family group that consists only of parents and children; joint family: where more than one generation live together in a common house; ^4^Currently smoke or using smokeless tobacco products such as tobacco leaf, Zarda, Gul etc.; ^5^blood pressure levels SBP ≥ 140 mmHg or DBP ≥ 90; ^6^25 kg/m^2^.

Table 2 shows descriptive statistics of health-related behaviors, comorbid conditions, and essential health service-related practices according to SES. Adverse health-related behaviors, such as non-adherence to recommended medication and diet was significantly higher among participants in the low-SES group than the high-SES group (*P* < *0.001*). Regarding non-adherence to necessary health service-related practices such as irregular visits to the diabetes center (*P* = *0.001*), not practicing self-monitoring of blood glucose (*P* = *0.00*2), and comorbid conditions, such as depressive symptoms (*P* < *0.001*) were significantly higher among participants in the low-SES group than the high-SES group. Adherence to physical activity (*P* = *0.009*) and proportion of non-overweight/obesity (*P* < *0.001*) were higher in the low-SES group than the high-SES group.

**Table 2:** Descriptive statistics of health-related behaviors, comorbid conditions, and essential health service-related practices according to SES among diabetes individuals: Bangladesh Diabetes study (*n* = 500).

Characteristics	SES, % (95% CI)			*P*-value^1^
	Low	Medium	High	
**Health-related behaviors**				
**Adherence to medication**				
No Yes	84.5 (84.3–84.7) 15.5 (15.2–15.7)	48.5 (37.8–59.3) 51.5 (40.7–62.2)	38.8 (27.8–51.1) 61.2 (48.9–72.2)	<0.001
**Adherence to diet**				
No Yes	81.6 (71.4–88.7) 18.5 (11.4–28.6)	77.3 (59.3–88.8) 22.8 (11.2–40.7)	63.0 (48.8–75.3) 36.9 (24.7–51.2)	<0.001
**Adherence to physical activity**				
No Yes	53.4 (31.2–74.6) 46.4 (25.4–68.8)	63.5 (46.9–77.4) 36.5 (22.7–53.1)	69.7 (65.9–73.3) 30.3 (26.8–34.1)	0.009
**Tobacco consumption**				
No Yes	84.5 (62.0–94.8) 15.5 (5.2–38.0)	88.6 (83.2–92.5) 11.4 (7.6–16.8)	87.3 (52.1–97.7) 12.7 (2.3–47.9)	0.528
**Comorbid conditions**				
**Hypertension**				
No Yes	60.1 (52.4–67.4) 39.9 (32.6–47.7)	55.7 (43.7–67.1) 44.3 (32.9–56.3)	52.7 (34.3–70.4) 47.3 (29.6–65.7)	0.392
**Overweight/obesity**				
No Yes	70.2 (57.0–80.7) 29.8 (19.2–43.0)	61.1 (59.1–63.1) 38.9 (36.9–40.9)	45.5 (25.6–66.9) 54.6 (33.1–74.4)	<0.001
**Depressive symptoms**				
No Yes	27.9 (19.2–38.9) 72.0 (61.1–80.9)	65.3 (50.8–77.4) 34.7 (22.6–49.2)	79.4 (70.2–86.3) 20.6 (13.7–29.8)	<0.001
**Essential health service-related practices**				
**Regular scheduled visit to diabetic center**				
No Yes	76.2 (51.2–90.7) 23.8 (9.3–48.8)	67.7 (46.2–83.6) 32.3 (16.4–53.8)	56.4 (44.8–67.3) 43.6 (32.7–55.2)	0.001
**Practicing self-monitoring of blood glucose**				
No Yes	71.4 (67.8–74.8) 28.6 (25.2–32.2)	61.1 (49.4–71.6) 38.9 (28.4–50.6)	52.7 (36.8–68.1) 47.3 (31.9–63.2)	0.002
**Rely on alternative medicine**				
No Yes	89.8 (84.3–93.6) 10.1 (6.4–15.7)	92.8 (79.0–97.8) 7.2 (2.2–21.0)	92.1 (77.4–97.5) 7.8 (2.5–22.6)	0.599

Note: ^1^ Man-Whitney U test were performed.

Table 3 presents the multivariate analysis of SES, health-related behaviors, comorbid conditions, and essential health service-related practices as potential mediators, and other sociodemographic factors associated with glycemic control. The rate of good glycemic control was four times (Adjusted odds ration [AOR] = 3.54, 95% confidence interval [CI] = 1.50–8.37) greater among individuals belonging to the high SES than low SES group. The relative odds of good glycemic control were three (AOR = 3.08, 95% CI = 1.47–6.45), two (AOR = 2.43, 95% CI 1.24–4.79), and three times (AOR = 2.91, 95% CI = 1.60–5.30) higher among participants who were adherent to their medications, diet, and physical activity than their counterparts.

**Table 3:** Adjusted odds ratio for associations between SES, other sociodemographic, health-related behaviors, comorbid conditions, and essential health service-related behavioral characteristics according to good glycemic control among diabetes individuals: Bangladesh Diabetes study (*n* = 500).

Characteristics	Achievement of good glycemic control	*P*-value
	Adjusted odds ratio (95% CI)	
**Sociodemographic**		
**Age, years**		
21–46 47–55 56–85	1.00 0.71 (0.36–1.37) 1.18 (0.58–2.39)	— 0.302 0.6520,
**Sex**		
Female Male	1.00 0.45 (0.24–0.82)	— 0.009
**Education**		
Higher secondary and above Secondary Primary No education	1.00 0.70 (0.24–2.03) 0.30 (0.09–0.96) 0.16 (0.04–0.59)	— 0.514 0.042 0.006
**Types of family structure**		
Nuclear Joint	1.00 1.57 (0.79–3.10)	— 0.198
**Currently married**		
No Yes	1.00 3.96 (1.12–14.03)	— 0.033
**Center**		
Rajshahi Barishal Dhaka	1.00 0.53 (0.27–1.04) 0.34 (0.17–0.69)	— 0.065 0.002
**Residence**		
Rural Urban	1.00 2.83 (1.47–5.44)	— 0.002
**Socioeconomic status**		
Low Medium High	1.00 1.10 (0.48–2.56) 3.54 (1.50–8.37)	— 0.821 0.004
**Duration of diabetes, years**		
<5 ≥5	1.00 0.43 (0.24–0.77)	— 0.004
**Health-related behaviors**		
**Adherence to medication**		
No Yes	1.00 3.08 (1.47–6.45)	— 0.003
**Adherence to diet**		
No Yes	1.00 2.43 (1.24–4.79)	— 0.010
Adherence to physical activity		
No Yes	1.00 2.91 (1.60–5.30)	— <0.001
**Tobacco consumption**^***4***^		
No Yes	1.00 0.83 (0.33–2.11)	— 0.701
**Comorbid conditions**		
**Hypertension**		
No Yes	1.00 0.61 (0.35–1.05)	— 0.077
**Overweight/obesity**		
No Yes	1.00 0.41 (0.23–0.75)	— 0.004
**Depressive symptoms**		
No Yes	1.00 0.42 (0.22–0.82)	— 0.011
**Essential health service-related practices**		
**Regular scheduled visit to diabetic center**		
No Yes	1.00 2.91 (1.58–5.36)	— 0.001
**Practicing self-monitoring of blood glucose**		
No Yes	1.00 4.86 (2.73–8.67)	— <0.001
**Rely on alternative medicine**		
No Yes	1.00 0.10 (0.02–0.51)	— 0.005

Overweight/obese and depressed patients were 0.41 (AOR = 0.41, 95% CI = 0.23–0.75) and 0.42 (AOR = 0.42, 95% CI = 0.22–0.82) times less likely to have good glycemic control than their counterparts. The odds of good glycemic control were three (AOR = 2.91, 95% CI = 1.58–5.36) and five times (AOR = 4.86, 95% CI = 2.73–8.67) higher among diabetic patients who were regularly visiting the diabetic center and self-monitored their blood glucose level. The relative odds of good glycemic control were 0.10 times lower (AOR = 0.10, 95% CI = 0.02–0.51) among participants who rely on alternative medicine than their counterparts.

Diabetic patients who were married and residence in an urban area were 3.96- and 2.83- fold more likely to have good glycemic control, respectively. The odds of good glycemic control were 0.34 (AOR = 0.34, 95% CI = 0.17–0.69), 0.45 (AOR = 0.45, 95% CI = 0.24–0.82), 0.43 (AOR = 0.43, 95% CI = 0.24–0.77) times lower among participants who were from Dhaka center, being male, and who had longer duration of diabetes (≥5 years), respectively.

Table 4 shows the direct and indirect effects in the applied model and percentage of mediation by the various potential mediators on the association of low SES and poor glycemic control. The mediation analyses showed a significant direct effect in the model of low SES and poor glycemic control with an AOR of 1.07 (95% CI = 1.05–1.10). The analyses also showed non-adherence to medication and diet, depression, irregular scheduled visits to the diabetes center, and not practicing self-monitoring of blood glucose level significantly mediate the relationship between low SES and poor glycemic control. The corresponding AORs were, 1.07 (95% CI = 1.04–1.13), 1.04 (95% CI = 1.02–1.06), 1.05 (95% CI = 1.04–1.09), 1.04 (95% CI = 1.03–1.06), and 1.05 (95% CI = 1.03–1.07), respectively.

**Table 4:** Direct and indirect effects (odds ratio scale) in the model of low SES on poor glycemic level operating via adverse health-related behaviors, comorbid conditions, and non-adherence to essential health service-related practices: Bangladesh Diabetes study (*n* = 500).

Potential mediators	Odds ratio	95% CI	% Mediated	95% CI
**Direct effect**	1.07	1.05, 1.10	20.3	16.8, 23.8
**Indirect effect of:**				
**Adverse health-related behaviors**				
Medication non-adherence	1.07	1.04, 1.13	20.2	16.7, 23.7
Dietary non-adherence	1.04	1.02, 1.06	12.0	9.2, 14.8
Tobacco consumption	1.01	0.99, 1.02	1.5	0.43, 2.6
**Comorbid conditions**				
Depressive symptoms	1.05	1.04, 1.09	15.0	11.9, 18.1
**Non-adherence to essential health service-related practices**				
Irregular scheduled clinic visits	1.04	1.03, 1.06	11.7	8.8, 14.5
Not practicing self-monitoring of blood glucose	1.05	1.03, 1.07	14.4	11.3, 17.5
Rely on alternative medicine	1.02	0.97, 1.03	4.9	3.0, 6.8
**Total indirect effect**	1.29	1.27, 1.32	79.7	76.1, 83.2
**Total effect**	1.38	1.21, 1.48		

Note: Model is adjusted for Age, sex, residence, education, center, family structure, and marital status.

Direct, indirect, and total effects are those calculated by Stata command *binary_mediation*.

Considering the total effect in the model of low SES on poor glycemic control, the direct effect in the model explained about 20.3% (95% CI = 16.8–23.8), whereas the remainder of the indirect effect in the model operating via non-adherence to medication and diet, depression, irregular scheduled visits to the diabetes center and not practicing self-monitoring of blood glucose level contributed 20.2%, 12.0%, 15.0%, 11.7%, and 14.4%, respectively, to the total effect of low SES on poor glycemic control. Overall, the AORs and their 95% CIs of total indirect effects in the model of low SES on poor glycemic control was 1.29 (95% CI = 1.27–1.32: Table 4).

## Discussion

To our knowledge, this is the first study in a developing country setting to formally examine mediation of the association between low SES and poor glycemic control through a complex range of health-related behaviors, comorbid conditions, and essential health service-related practices, and to determine the extent to which these potential modifiable factors were involved in this relationship in diabetics in Bangladesh. The results of the present study showed that SES inequalities prevent the achievement of good glycemic control among type 2 diabetics in Bangladesh, where patients with low SES have poorer glycemic control than those with higher SES. This study also suggested that the association between low SES and poor glycemic control are mediated by non-adherence to recommended medication and diet, through non-adherence to essential health service-related practices concerning diabetes care, such as irregular scheduled visits to the diabetes center and not practicing self-monitoring of blood glucose concentrations, and finally through depressive symptoms. Identified modifiable factors substantially explained the relationship between low SES and poor glycemic control (79.7%).

The results of the present study indicated that 28.2% of people with diabetes in Bangladesh had good glycemic level control. A similar low prevalence of good diabetes control was also identified in other previous small-scale studies (18–28.6%)^38,39^ and a nationwide survey (13%)^3^ in this country. This high prevalence of poor glycemic control is a matter of urgent concern in Bangladesh because of its negative health implications for patients.

Our findings were consistent with those of previous studies indicating that female patients, patients in urban areas, and patients who were married tended to achieve better control^7,38^ and that longer duration of diabetes^40,41^ and non-adherence to recommended medication, diet, and physical activity^4,7,25,41^ were associated with poor control. Similar to previous reports, our findings showed that regular scheduled visits to the diabetes center and practicing self-monitoring of blood glucose level^21,42^ were associated with good glycemic control, and that relying on alternative medicine and being overweight/obese or suffering from depressive symptoms were associated with poor control.

Diabetic individuals belonging to the low-SES group had poorer glycemic control than those with higher SES in the present study, consistent with some previous studies conducted in both developed^6,25^ and developing countries^3,4,6^. These results may provide insight into the development of means to improve glycemic control and reduce the incidence of chronic complications among diabetics with low SES.

The identified modifiable factors, particularly adverse health-related behaviors, such as non-adherence to medication, was responsible for a greater proportion of the association between low SES and poor glycemic control in our study, in contrast to a previous study conducted in Canada^24^. This discrepancy may have been attributable to the differences in metrics used to assess medication non-adherence and/or differences in the health care setting and SES. However, our findings agreed with other studies^43,44^ showing that low SES was associated with non-adherence to medication. In Bangladesh, along with a lack of knowledge and illiteracy, out-of-pocket medication costs pose a significant burden to access to medication among low-SES patients. Studies based on survey data demonstrated that poorer households had higher risks of catastrophes than wealthier households in terms of chronic illness management^45,46^.

Consistent with the Canadian study^24^, our results showed that dietary non-adherence was a significant intermediary of the association between low SES and poor glycemic management. Jaffiol *et al*.^47^ reported that low SES was associated with undesirable diabetes meal patterns. People within the low SES cluster consumed large amount of carbohydrates and fewer super molecules, vegetables, and contemporary fruits according to their financial affordability. Therefore, information regarding access to healthy foods with reasonable cost and methods of preparing meals at home inexpensively are useful if they are provided targeting to patients living under low SES environments. This information will help the patients with limited income in Bangladesh to be adherent to dietary advice regardless of any economic difficulties they face.

Among the comorbid conditions examined, depressive symptoms were shown to significantly intervene the association between low SES and poor glycemic control. Individuals with diabetes are more possible to suffer from depression than non-diabetic individuals, and the incidence of depression as a comorbidity with diabetes decreased with higher SES^48^. Low-SES patients struggling with depressive symptoms may no longer be willing or able to adopt self-management behaviors^48^, which may lead to poorer glycemic control. These findings highlighted the importance of regular screening of diabetic patients for depressive symptoms, especially among patients from lower SES groups, and to provide appropriate treatment to such patients.

Among the factors related to the non-adherence to essential health service-related practices concerning diabetes care, irregular scheduled visits to the diabetes center mediated the relationship between low SES and poor glycemic control. Adherence to the suggested schedule of visits to clinics (in Bangladesh, visits at least every 3 months were recommended for patients with diabetes)^29^ is critical for diabetes control^21^. Similar to many other observational studies^47,48^, we also found that adherence to treatment plans was lower among patients with low SES, leading to poorer diabetes control. Speculated common reasons for irregular clinic visits among diabetes patients in the lower SES cluster in Bangladesh might be difficulties to understand importance of adhering to diabetes treatment, cost burden associated with treatment, lack of family support, costly and complex transportation system, and a lack of health information^49^. Further studies are needed to gain insight into reasons for irregular health care use among patients living in low SES environment.

Among the essential health service-related practices examined, practicing self-monitoring of blood glucose at home or at the pharmacy, which significantly mediated the relationship between low SES and poor glycemic control, was of particular importance. Self-monitoring of blood glucose concentration is regarded a crucial component of diabetes care by the American Diabetes Association^42^, because in contrast to the testing of HbA1c levels, self-monitoring allows for greater scrutiny of daily glucose fluctuations. Similar to other studies^50,51^, the results of this study also indicated that diabetics with lower SES were less likely to practice self-monitoring and be in good control of their HbA1c level. In spite of the fact that blood glucose self-monitoring equipment and supplies are accessible in Bangladesh, diabetic patients, particularly those with lower SES, are incapable to secure the equipment and testing strips for their regular use. In Bangladesh, the average cost of using a glucose meter per year was calculated as US$ 117.3 based on an assumed glucose meter life of 3 years^52^. This may be of a disproportionate financial burden for the patients with low SES to practice self-monitoring of blood glucose, regularly.

There are some limitations in this study. First, data used in the analysis were cross-sectional information, therefore, the results did not allow us to discuss longitudinal mediation mechanism. The cross-sectional results do not provide information on directions of the relationship; therefore, causal mechanisms are not answered yet. For example, it is conceivable that depressive symptoms may have driven to lower SES. Future longitudinal research by following a cohort will provide information to discuss the mediators influencing on the relationship of low SES at the baseline and glycemic control in the follow up phase.

Second, the obtained results are generally applicable to urban population because diabetes clinics were located in urban areas and 63.2% of the participated patients in our study were living in urban areas.

Third, our study subjects were type 2 diabetes patients without using insulin. Patients using insulin are generally on the treatment for longer years, which is a risk factor for non-compliance with therapy recommendations and with poor glycemic control. Although the prevalence of insulin monotherapy among Bangladeshi type 2 diabetes patients accounts for less than 7.3% to 14.0%^53,54^, further studies focusing on this group are required to provide evidence-based recommendations to maintain glycemic control for long-term diabetes patients in Bangladesh.

Forth, most of the data used in the analysis, except blood examination and physical measurement, were self-reported. Although, the results should be carefully interpreted assuming potential recall bias, the analyses were performed based on the assumption that there are reasonably accurate estimates of adherence by self-reported questionnaire^55^. To minimize recall bias, the face-to-face interview methodologies instead of self- administered questionnaire, and a 7-day recall period was applied for their answers.

Fifth, this study did not analyze medication regimen complexity. Although some studies address its influence on glycemic control^55,56^, a lack of information on other than anti-diabetic mediation and data on details of regimen restricted analysis. Access to over the counter (OTC) medication was also not evaluated. These issues may have over- or underestimated participants’ true regimen complexity while calculating adherence to medication.

Sixth, household wealth was evaluated indirectly by developing an asset-based index, because of insufficiency reliable household economic indicators across households of all social categories in low-and middle-income countries. An asset-based index is mostly treated as a decent proxy for house economic standing. Lastly, there may have been selection bias, as this study was conducted only among participants who visited the outpatient diabetes clinics of hospitals located in urban areas. Although, the findings are only based on information from people who had access to healthcare services for diagnosed of type 2 diabetes in Bangladesh, the study subjects are selected from patients attending diabetes centers, where most of the diagnosed patients are registered regardless of sociodemographic, illness, and treatment characteristics. Further population-based studies by sampling subjects from the community are warranted.

This study had three significant strengths. First, this was the first study to formally examine factors mediating low SES-poor glycemic control association through a complex extend of health-related behaviors, comorbid conditions, and essential health service-related practices. Second, we used HbA1c value to assess glycemic control, which is a robust measure. Third, the random selection of multicentric analysis indicated higher external validity of this study.

## Conclusions

The present study provided further evidence of low SES-poor glycemic control association, and showed that adverse health-related behaviors, such as non-adherence to medication and diet, existing comorbid conditions such as depressive symptoms, and non-adherence to essential health service-related practices such as irregular scheduled clinic visits and not practicing self-monitoring of blood glucose concentration at home, represent pathways between social adversity and poor glycemic management. Those modifiable factors mediating the association between low SES and poor glycemic control in urban areas of Bangladesh and provide useful information for developing interventions to mitigate socioeconomic disparities in glycemic control. Further studies in patients living in the community as well as inclusion of individuals with type 2 diabetes with insulin monotherapy are warranted.

## Data Availability

The datasets used and/or analyzed during the current study available from the corresponding author on reasonable request.
